# Rap2b siRNA significantly enhances the anticancer therapeutic efficacy of Adriamycin in a gold nanoshell-based drug/gene co-delivery system

**DOI:** 10.18632/oncotarget.15508

**Published:** 2017-02-19

**Authors:** Li Ding, Ruonan Sun, Xinyue Zhang

**Affiliations:** ^1^ College of Bioscience and Biotechnology, Yangzhou University, Yangzhou, Jiangsu 225009, China; ^2^ Institute of Comparative Medicine, Yangzhou University, Yangzhou, Jiangsu 225009, China; ^3^ Jiangsu Co-innovation Center for Prevention and Control of Important Animal Infectious Diseases and Zoonoses, Yangzhou, Jiangsu 225009, China; ^4^ Jiangsu Key Laboratory of Zoonosis, Yangzhou, Jiangsu 225009, China; ^5^ Joint International Research Laboratory of Agriculture and Agri-Product Safety, Yangzhou University, Yangzhou, Jiangsu 225009, China

**Keywords:** Rap2b, siRNA, Adriamycin, gold nanoshells, cancer therapy

## Abstract

Rap2b is a novel p53 target we have identified recently. Knockdown of Rap2b sensitizes HCT116 cells to adriamycin-induced apoptosis, indicating that Rap2b promotes adriamycin resistance in cancer cells. In the present study, we designed a nanostructure-based drug/gene delivery system to evaluate the potential of Rap2b siRNA as a therapeutic agent against human cancers. Specifically, after co-incubated with HCT116 cells, adriamycin- and Rap2b siRNA-loaded gold nanoshells were internalized. Subsequent laser irradiation promoted release of adriamycin and Rap2b siRNA from the nanoparticles. The laser-induced release of Rap2b siRNA decreased cellular expression of Rap2b and significantly enhanced the anticancer therapeutic efficacy of adriamycin *in vitro* and *in vivo*. In addition, laser irradiation of the nanoparticles might exert an additional thermal killing effect on cancer cells and further improved the anticancer efficacy of adriamycin. In summary, Rap2b siRNA is a potential enhancing agent for adriamycin-based anticancer therapeutics and the gold nanoshell-based drug/gene delivery system carrying both adriamycin and Rap2b siRNA provides a promising anticancer therapeutic strategy.

## INTRODUCTION

Tumor suppressor p53 is identified as a DNA sequence-specific transcription factor and a stress sensor [1]. Upon various stresses, such as DNA damage, p53 is activated and then activates or represses numerous downstream genes. These genes elicit various cellular outcomes, such as cell cycle arrest, DNA repair, and apoptosis, which lead to tumor inhibition [1–4]. However, p53 also activates certain target genes which in turn help cancer cells survive, such as Hzf and IRF2BP2 [5, 6]. Rap2b, a novel p53 target we have identified recently, is also such a gene [4]. Rap2b predominantly upregulates in many types of human tumors (∼80%). Down-regulation of Rap2b sensitizes HCT116 colorectal cancer cells to apoptosis induced by adriamycin (Adr), indicating that Rap2b promotes Adr resistance in cancer cells [4]. These data encourages us to establish a dual drug/gene delivery system and co-deliver both Rap2b siRNA (siRap2b) and Adr into cancer cells to enhance the anticancer therapeutic efficacy of Adr.

As we know, nanoscale particles show numerous advantages over conventional formulations in anticancer therapeutics because of its unique outstanding magnetic, photoelectric, and photothermal properties [7, 8]. To our best knowledge, gold nanoparticles (GNPs) are mostly adapted to biomedical applications among numerous nanoscale materials [9–11]. You, *et al*. have reported that Adr-loaded hollow GNPs shows enhanced cytotoxic effects *in vitro* and *in vivo* [12]. Moreover, Huschka, *et al*. have confirmed that gold nanoshells and nanorods are excellent nucleic acid carriers for gene therapy [13]. In addition, spherical nucleic acid nanoparticle conjugates (SNAs) consist of densely packed siRNA oligonucleotides surrounding an inorganic GNP core [14–18] and form a platform for gene silencing. SNAs act as single-entity agents capable of simultaneous transfection and regulation of genes with no need for auxiliary carriers or cationic transfection agents. Furthermore, SNAs are remarkably stable in physiological environments and resistant to nuclease degradation. Compared to conventional RNA interference (RNAi) delivery platforms, SNAs provide a more efficient and long-standing *in vivo* knockdown of genes without triggering a significant immune response and off-target effect [19–21].

Due to the superior features of GNPs, we designed a drug/gene co-delivery system for oncotherapy in this study using a gold nanoshell (GN), a member of GNP family with a hollow structure [12, 22–24]. The chemically inert and non-toxic GNs [9] enable a drug/gene accumulation in tumors via an enhanced permeability and retention (EPR) effect [25]. After PEGylation, the GNs possess an enhanced circulation half-life *in vivo* [26, 27]. Moreover, GN particles strongly absorb near infrared ray (NIR), which induces photothermal energy conversion. The converted energy can locally heat nanoscale volumes instead of the bulk of solution volume [28]. This phenomenon is commonly referred to as photothermal heating [23, 29] and can be used to induce drug release from GNs [30] and localized hyperthermia to kill cancer cells [31].

**Figure 1:**
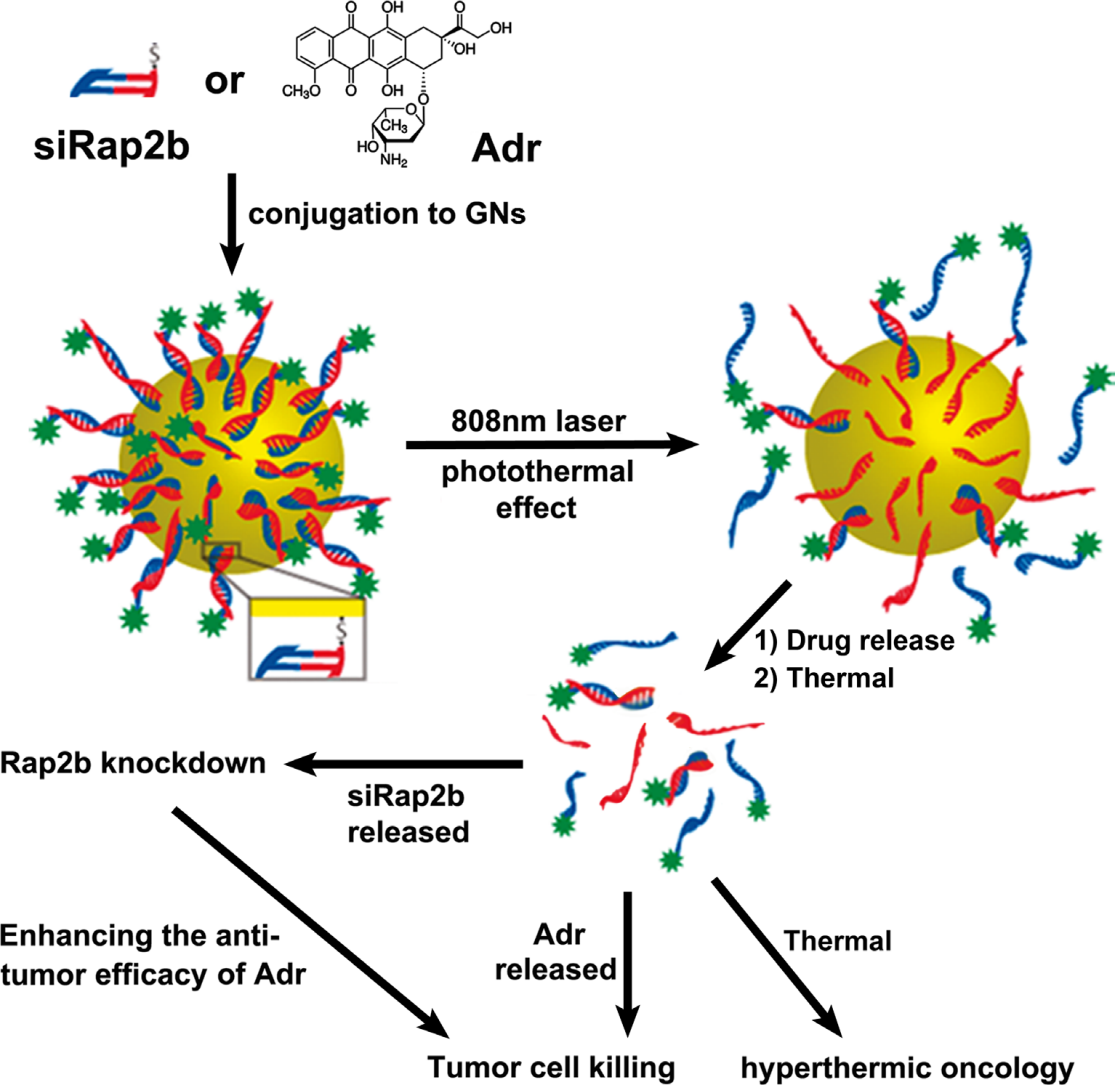
The design of this study Adr and synthesized siRap2b were chemically conjugated to GNs, respectively. Subsequently, drug-loaded GNs were treated with an 808 nm laser. The laser treatment generated a photothermal effect, which greatly accelerated drug release. The released Adr killed cancer cells directly. Moreover, the released siRap2b significantly decreased the expression of Rap2b and thus leaded to an enhanced anticancer therapeutic efficacy. In addition, laser-induced thermal effect exerted a direct thermal killing effect on cancer cells/tissues.

As shown in Figure 1, in this study, we synthesized PEGylated GNs, to which Adr and siRap2b molecules were conjugated, respectively. The conjugates were then co-cultured with cancer cells or injected into tumor-bearing mice. As expected, regardless of conjugation to GNs, siRap2b significantly down-regulated the expression of Rap2b in cancer cells. The most notable observation in this study is that siRap2b greatly enhanced the anticancer efficacy of Adr. Specifically, when irradiated with a NIR laser, the GN complex released more Adr and siRap2b molecules and leaded to an increased anticancer therapeutic efficacy. In addition, laser irradiation might also exert an additional thermal killing effect on cancer cells. Taken together, our results revealed that siRap2b significantly enhanced the anticancer therapeutic efficacy of Adr and GN-based co-delivery of siRap2b and Adr generated a promising anticancer therapeutic strategy.

## RESULTS

### Preparation of GNs, Adr-GNs and siRap2b-GNs

**Figure 2:**
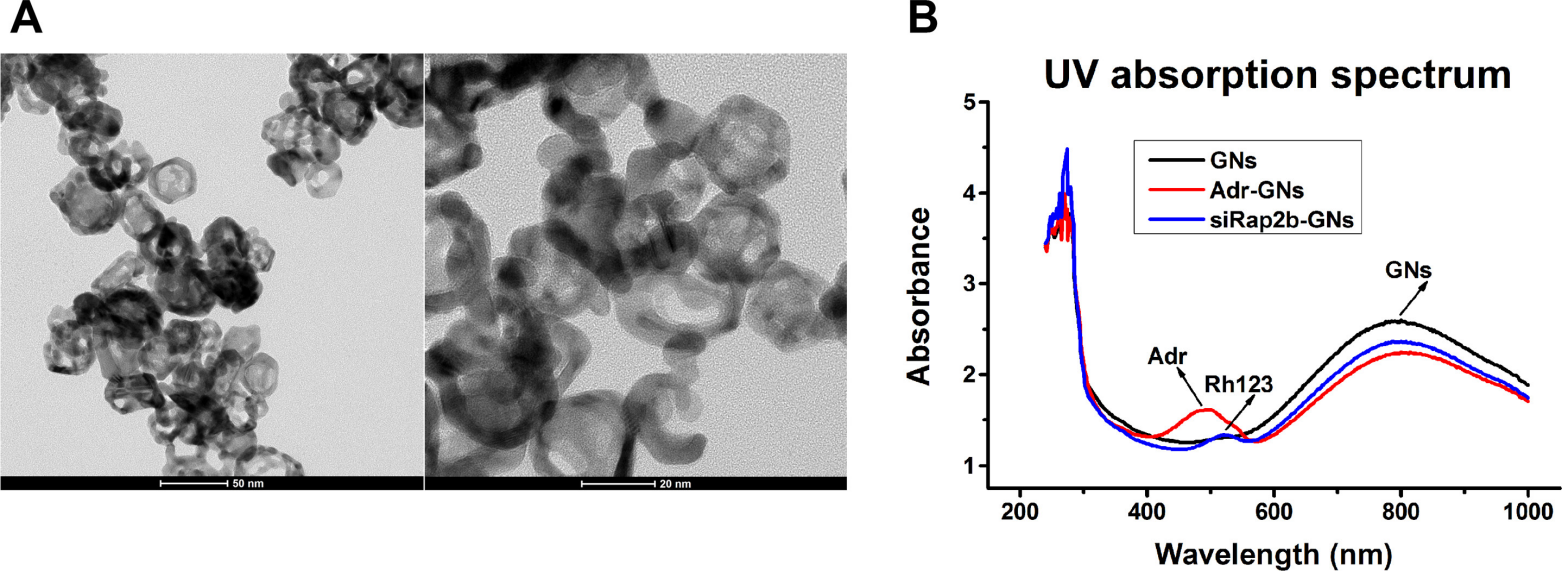
Characterization of PEGylated GNs and derivatives (**A**) Transmission electron microscope images of PEGylated GNs. (**B**) The UV absorption spectra of GNs, Adr-GNs, and siRap2b-GNs.

To prepare the GNs, we employed a common approach by reducing HAuCl_4_ onto silver nanoparticles [12] and then PEGylated the freshly prepared GNs. As shown in Figure 2A, the PEGylated GNs were characteristic of a hollow structure with an average diameter of ∼28 nm and an absorption peak at 786 nm (Figure 2B).

Subsequently, we successfully conjugated Adr and siRap2b molecules to the PEGylated GNs (Adr-GNs, siRap2b-GNs), as shown in Figure 2B. The absorption peak at 500 nm demonstrated that Adr molecules were conjugated to the GNs. However, due to high background absorption of GNs at 240–300 nm, the absorption peak for siRap2b could not be clearly identified from the UV spectrum of siRap2b-GNs. Therefore, we labeled siRap2b with Rhodamine 123 (Rh123: excitation, 507 nm; emission, 529 nm), a green-fluorescent dye, at the 3′ end of the sense chain. As shown in Figure 2B, the absorption peak at 522 nm indirectly exhibited a successful conjugation of siRap2b to GNs. On average, each GN particle was loaded with 6 × 10^4^ Adr or 200 siRap2b molecules.

### Laser-induced thermal effect and drug release

**Figure 3:**
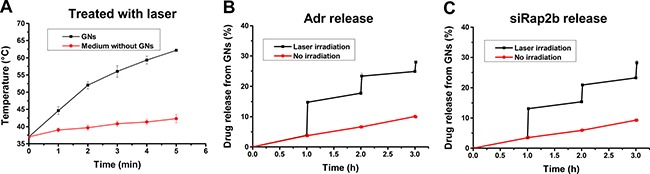
Laser-induced thermal effect of GNs and drug release GNs were dissolved in McCoy's 5A medium, placed in a 37°C waterbath, and treated with an 808 nm laser (2 W·cm^−2^). (**A**) The laser-induced temperature increase at indicated irradiation time points with GN-free McCoy's 5A medium as a control. (**B**) The release of Adr from Adr-GNs after a laser treatment for 1 min at 1 h, 2 h, and 3 h, respectively. (**C**) The release of siRap2b from siRap2b-GNs after a laser treatment for 1 min at 1 h, 2 h, and 3 h, respectively. Results are expressed as mean ± standard deviation from 3 independent experiments.

Laser irradiation can induce photothermal energy conversion and thus heat a GN solution. As shown in Figure 3A, upon exposure to an 808 nm laser (2 W·cm^−2^), the temperature of the GN solution (pre-warmed in a 37°C water bath) gradually increased to 44.7°C and 62.2°C at 1 min and 5 min post irradiation, respectively. Moreover, upon laser irradiation, the temperature of the GN solution increased much faster than that of a GN-free McCoy's 5A medium (*P* < 0.01).

To evaluate laser-induced drug release of GN particles, we exposed the cells under an 808 nm laser (2 W·cm^−2^) for 1 min to control the temperature under 45°C according to the results in Figure 3A. Then, we measured drug release efficiency in a 37°C water bath. As shown in Figure 3B, 3C, without laser irradiation, the GN complex released Adr or siRap2b slowly to the solution. Upon laser exposure, the release efficiency increased sharply (*P* < 0.01). These data provide us a guideline for the *in vivo* study on laser-induced drug release.

### Cellular uptake of Adr-GNs and siRap2b-GNs

**Figure 4:**
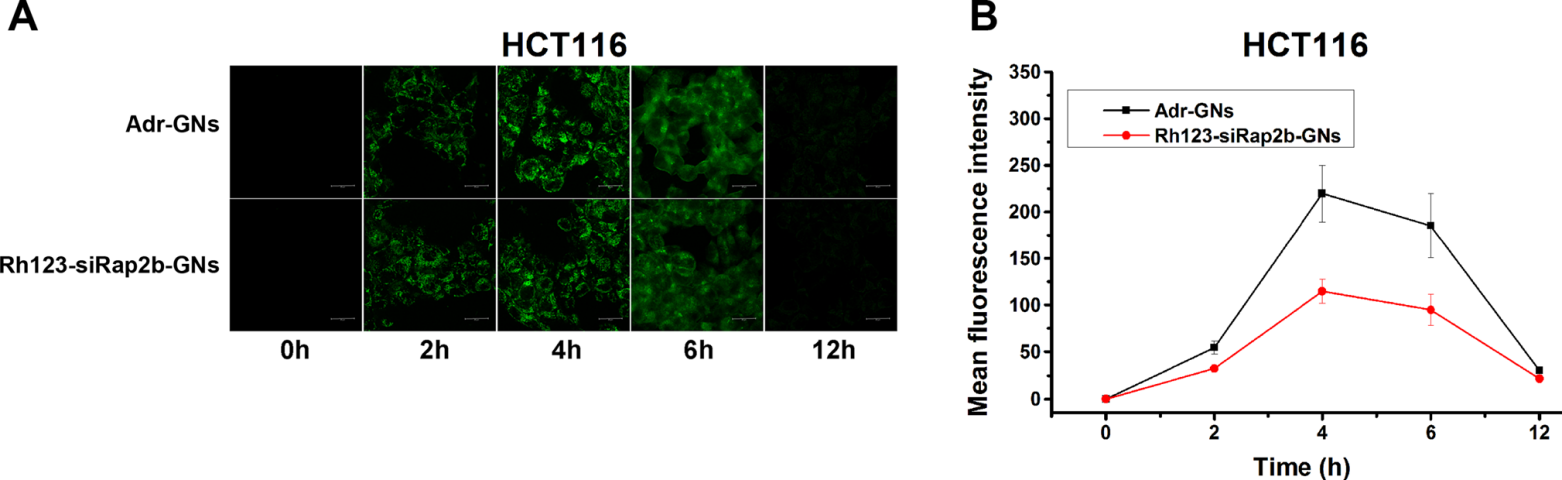
Cellular uptake of drug-loaded GNs (**A**) Visualization of cellular uptake of Adr-GNs and Rh123-siRap2b-GN by HCT116 cells at indicated time points under a laser scanning confocal microscope. (**B**) Mean fluorescence intensity of HCT116 cells after incubated with Adr-GNs and Rh123-siRap2b-GNs at different time points. Results are expressed as means ± standard deviation (*n* = 10).

To measure the maximum cellular accumulation time of GNs, uptake of Adr-GNs and siRap2b-GNs were carried out in HCT116 cells. As shown in Figure 4, fluorescence intensity showed a similar dynamics in the cells incubated with Adr-GNs or Rh123-labeled siRap2b-GNs (Rh123-siRap2b-GNs). The fluorescence intensity increased in the beginning, reached a maximum at 4 h, and then gradually decreased to an undetectable level.

### Rap2b knockdown

**Figure 5:**
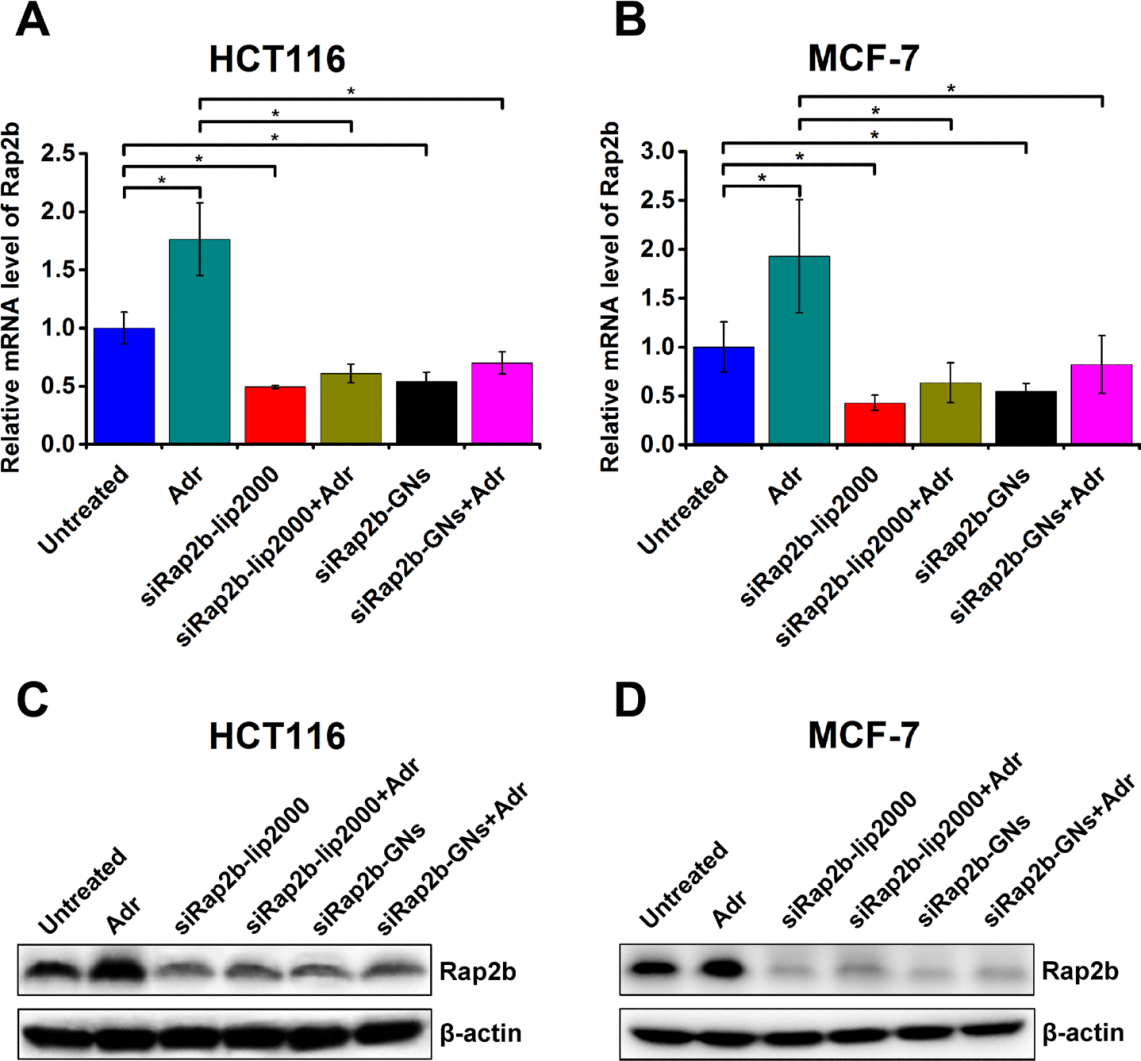
Knockdown of Rap2b in human cancer cells HCT116 and MCF-7 cells were treated as indicated in the main text and harvested for the analysis of Rap2b knockdown. (**A**–**B**) Real-time PCR analyses to evaluate the relative mRNA levels of Rap2b in HCT116 (A) and MCF-7 (B) cells with various treatments. 28S RNA was used as an internal control to normalize the data. Error bars represent means ± standard errors of the mean. *P < 0.05, *n* = 3. (**C**–**D**) Western blot analyses to evaluate the protein levels of Rap2b and β-actin in HCT116 (C) and MCF-7 (D) cells of different treatment groups.

To evaluate the knockdown efficiency of Rap2b caused by siRap2b-GNs, we performed quantitative real-time polymerase chain reaction (qRT-PCR) and western blot analyses. The mixture of siRap2b and Lipofectamine^®^ 2000 (siRap2b-lip2000) was used as an active control. The results demonstrated that when compared with untreated controls, siRap2b-GNs successfully down-regulated the mRNA (Figure 5A and 5B) and protein (Figure 5C and 5D) levels of Rap2b in both HCT116 and MCF-7 cells. Moreover, the knockdown efficiency caused by siRap2b-GNs was similar to that by siRap2b-lip2000. In addition, down-regulation of Rap2b expression was also evaluated using the cells treated with Adr (1 μM, 8 h), a genotoxic chemical that results in an increase in the expression of Rap2b [4]. When treated with siRap2b-GNs+Adr, both HCT116 and MCF-7 cells successfully maintained significantly low levels of Rap2b mRNA (Figure 5A, 5B) and protein (Figure 5C, 5D), which was similar to that in the siRap2b-lip2000+Adr controls. Collectively, siRap2b-GNs treatment successfully down-regulated the expression of Rap2b in the absence and presence of Adr, indicating the applicability of GNs for siRNA delivery in this study.

### Enhanced role of siRap2b in killing cancer cells by Adr *in vitro*

**Figure 6:**
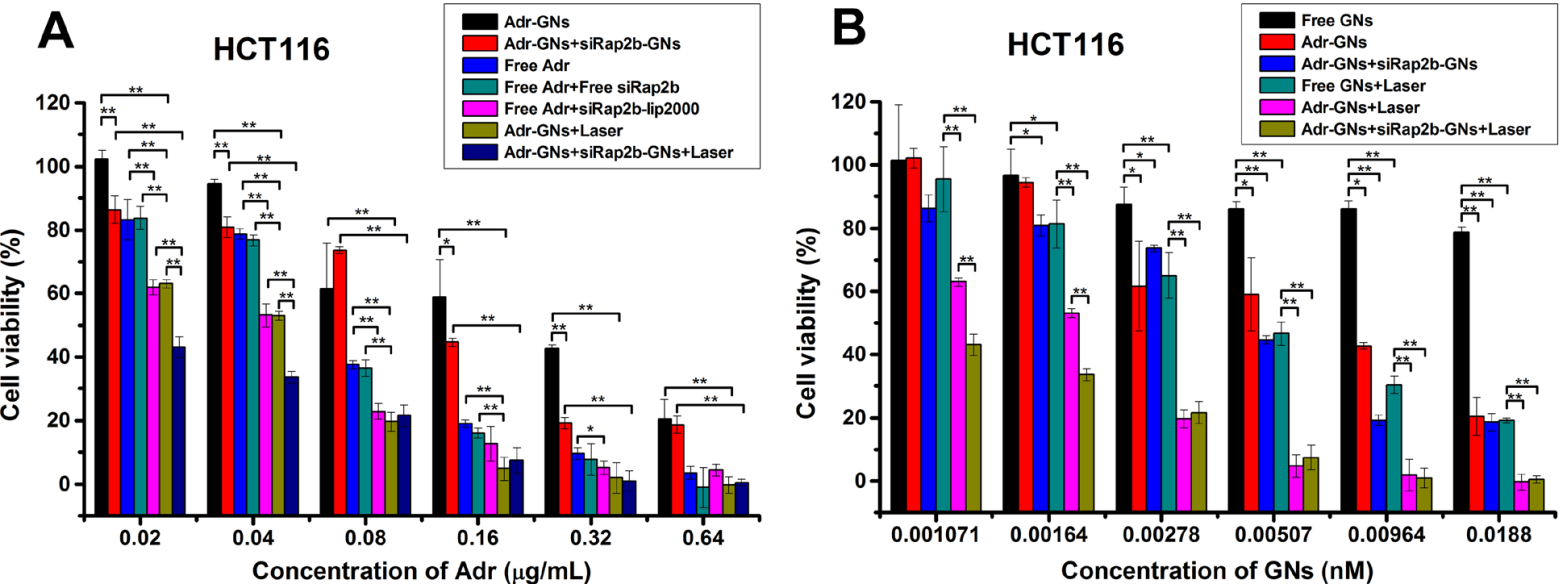
*In vitro* killing effect of various treatments on HCT116 cells Cells were treated with an 808 nm laser (2 W·cm^−2^) for 1 min as indicated. (**A**) Analyses of the killing effect of various treatments on HCT116 cells with Adr concentration as an independent variable. (**B**) Analyses of the killing effect of various treatments on HCT116 cells with GN concentration as an independent variable. Error bars represent means ± standard deviation. *P < 0.05, **P < 0.01, *n* = 4.

To evaluate the efficacy of siRap2b in killing cancer cells by Adr *in vitro* in a GN-based platform for drug delivery, we first performed an MTT (methylthiazolyldiphenyl-tetrazolium bromide) assay. As shown in Figure 6A, Free Adr treatment resulted in a reduction in the cell viability in a concentration-dependent manner, which was similar to the results reported by Li *et al*. [32]. However, combination of Free Adr and siRap2b-lip2000 resulted in a significant decrease in cell viability in a lower concentration of Adr (≤ 0.32 μg/mL) (*P* < 0.05). Similarly, when we conjugated Adr and siRap2b molecules to GNs, Adr-GNs+siRap2b-GNs treatment showed a stronger killing effect than Adr-GNs treatment. Moreover, when we further exposed Adr-GNs and Adr-GNs+siRap2b-GNs treated HCT116 cells to an 808 nm laser (2 W·cm^−2^) for 1 min, we observed an enhanced reduction in cell viability (*P* < 0.01). Specifically, Adr-GNs+Laser treatment displayed a capability to kill cancer cells similar to that caused by Free Adr+siRap2b-lip2000 treatment, and superior to that caused by Free Adr or Free Adr+Free siRap2b treatments (*P* < 0.05). Furthermore, additional siRap2b-GNs enhanced the capability of Adr-GNs+Laser to reduce cancer cell viability in lower concentrations of Adr (0.02 and 0.04 μg/mL) (*P* < 0.01), indicating the highest killing effect on HCT116 cells in this study.

Next, we analyzed the cellular killing effect based on the concentration of GNs. As shown in Figure 6B, Free GNs didn't show significant cytotoxicity to HCT116 cells in all GN concentrations we tested. This is consistent with the common view that in general, Au-based nanoparticles are well tolerated [12]. Moreover, loading of Adr endowed the GNs with a cellular killing effect when the GN concentration ranged from 0.00278 nM to 0.0188 nM (*P* < 0.05), whereas loading of both Adr and siRap2b onto GNs showed a better killing effect with a broader GN concentration ranging from 0.00164 nM to 0.0188 nM (*P* < 0.05). Furthermore, upon laser illumination, GNs showed an obvious killing effect on HCT116 cells with concentrations ranging from 0.00164 nM to 0.0188 nM (*P* < 0.05). Loading of Adr enhanced the cellular killing effect of GNs+Laser with GN concentrations ranging from 0.001071 nM to 0.0188 nM (*P* < 0.01), whereas loading of both Adr and siRap2b further enhanced the killing effect of Adr-GNs+Laser treatment even with a lower GN concentration ranging from 0.001071 nM to 0.00164 nM (*P* < 0.01).

Taken together, our *in vitro* results indicated that siRap2b enhanced the killing effect of Adr on HCT116 cells and the most efficient strategy was a combination of Adr-GNs and siRap2b-GNs coupling with laser irradiation.

### Enhanced role of siRap2b in treating HCT116 tumors with Adr *in vivo*

**Figure 7:**
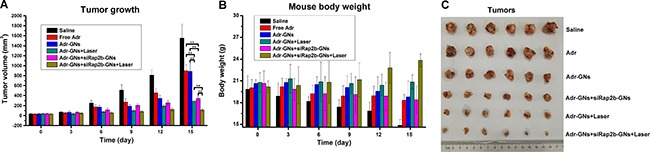
Efficacy of various therapeutic strategies in treating nude mice bearing HCT116 tumors Animals were treated with an 808 nm laser (2 W·cm^−2^) for 1 min as indicated. (**A**) Mean tumor volumes in various treatment groups. (**B**) Mean mouse body weights in various treatment groups. (**C**) Tumors resected from mice in various treatment groups. Error bars represent means ± standard deviation. *P < 0.05, **P < 0.01, *n* = 6.

To explore the potential of siRap2b for anticancer therapeutics, we evaluated the enhanced role of siRap2b in the Adr treatment of HCT116 tumors in a nude mouse model. In this study, a GN-based platform was used for drug delivery. As shown in Figure 7A, 7C, the tumors in the saline treated mice grew faster than those of the mice in any other treatment (*P* < 0.05). The tumor volume was reduced by 43% at 15 days post administration of Adr-GNs, which was similar to that in the Free Adr group (∼42%). However, after laser irradiation, the tumors in the Adr-GNs+Laser group was reduced by 82% in mean volume, exhibiting a more significant decrease than that in the non-irradiation group (*P* < 0.01). As expected, the mean tumor volume in the Adr-GNs+siRap2b-GNs treated mice was reduced by 78%, demonstrating a significant improvement in the therapeutic efficacy over the Adr-GNs group (*P* < 0.01). Laser treatment further enhanced the tumor inhibition effect of Adr-GNs+siRap2b-GNs with a 93% reduction in the tumor volume, indicating the highest inhibition efficiency among all treatment groups (*P* < 0.01).

Furthermore, the mean body weight of mice bearing HCT116 tumors continually increased in the Adr-GNs+siRap2b-GNs+Laser group during the 15 d treatment period (Figure 7B), whereas a significant decrease was observed in the mean body weight (20 g to 14 g) of the saline treated group (*P* < 0.05) (Figure 7B). For other groups of mice, the mean body weight only slightly increased or decreased during the whole treatment period (*P* > 0.05). All animals survived through the whole 15 d treatment period and no significant toxic side effect was observed in the *in vivo* therapeutic study.

Together, our *in vivo* results demonstrated that siRap2b enhanced the anticancer therapeutic efficacy of Adr in a nude mouse model and Adr-GNs+siRap2b-GNs+Laser treatment was the most efficient therapeutic strategy compared with others in this study.

## DISCUSSION

There are two major problems in cancer chemotherapy, toxic side effect and multidrug resistance (MDR). Nanoparticle-based drug delivery can overcome these problems via tumor targeting and MDR circumvention [33–35]. Basically, MDR falls into two distinct categories, pump and nonpump resistances [34, 36]. The major mechanism of nonpump resistance development is via activation of cellular anti-apoptotic defense, which is mainly mediated by oncogenes, such as *bcl-2*. Currently, targeting nonpump resistance is one of the important strategies in anticancer therapeutics. As reported by Alex, *et al*. [37], co-delivery of Adr and bcl-2 siRNA by nanoparticles successfully enhanced the efficacy of chemotherapy against multidrug-resistant cancer cells. Recently, we have reported that Rap2b possesses a pro-survival function in cancer cells [4], indicating that Rap2b is a nonpump resistance gene and a promising anticancer target.

As we know, RNAi-based gene silencing has emerged as a promising approach in anticancer therapeutics. However, many factors limit RNAi-based therapeutics in the clinical practice. These factors include lack of efficient drug delivery, various enzymes promoting RNA degradation in the systemic circulation, limited biological activity, and unfavorable safety profile [38]. Nanotechnology has provided feasible solutions for these problems. Recently, tumor targeting capability of GNs has been confirmed in numerous studies [31, 39, 40]. It has been reported that SNAs can form a unique micro-environment that inhibits enzymatic degradation of nucleic acids and thus results in an increased stability of siRNAs [14, 15, 20] and potentially longer therapeutic lifetimes. Moreover, scientists have used SNAs as an effective RNAi-based therapeutic agent for glioblastoma [16, 19, 21]. In this study, we designed a nanoparticle-based drug/gene co-delivery system to evaluate the potential of siRap2b as an anticancer therapeutic agent.

In the current study, the GNs were coated with PEG via thiol-Au bonds, and then Adr and siRap2b molecules were conjugated to GNs via amino-Au and thiol-Au bonds, respectively. Since an amino-Au bond is weaker than a thiol-Au one, excessive PEGylation will competitively inhibit abundant Adr from bonding to GNs. Therefore, we slightly modified the reported protocol to achieve a reasonable drug loading rate. Our data demonstrated that GN particles could be functionalized to deliver both Adr and siRap2b into cancer cells. The internalized siRap2b successfully decreased the expression of Rap2b and enhanced the anticancer therapeutic efficacy of Adr. Moreover, laser irradiation leaded to a local increase in temperature (Figure 3A) and thus greatly accelerated cellular release of Adr (Figure 3B) and siRap2b (Figure 3C), which further improved the therapeutic efficacy. In addition, we optimized the laser treatment condition and exposed the cells to an 808 nm laser irradiation (2 W·cm^−2^) for 1 min in order to control the temperature under 45°C. Under such a condition, thermal killing effect may not be apparent. Therefore, we can reasonably evaluate the role of siRap2b in enhancing the anticancer therapeutic efficacy of Adr. Furthermore, as shown in Figure 5, there is space to improve the knockdown efficiency of Rap2b. Predictably, if we can further modify the protocol to conjugate more siRap2b molecules to GNs, we will obtain a better knockdown efficiency and thus a better anticancer therapeutic efficacy. Therefore, one future direction is to optimize the conjugation protocol so as to improve the loading efficiency of siRap2b. Furthermore, as shown in Figure 6, there was no obvious difference in cell viability inhibition between Adr-GNs+siRap2b-GNs+laser and Adr-GNs+laser when the concentration of Adr was ≥ 0.08 μg/mL. It might be that the higher concentrations of Adr are sufficient to kill cancer cells. Thus, another future direction is whether siRap2b can also enhance the therapeutic efficacy of Adr against Adr resistant cancer cells.

Encouraged by the exciting *in vitro* killing effect of Adr- and siRap2b-loaded GNs on cancer cells, we further performed an animal study to evaluate the therapeutic efficacy *in vivo*. Overall, the *in vivo* and *in vitro* results showed a similar trend of anticancer therapeutic efficacy. Interestingly, in the *in vivo* study, the anticancer efficacy of Adr-GNs was similar to that of Free Adr, whereas in the *in vitro* study, the killing effect of Adr-GNs was lower than that of Free Adr (*P* < 0.05). This discrepancy mainly results from the tumor targeting capability of GNs caused by an EPR effect [31, 39, 40], which allows nanoparticles to passively accumulate in tumors [26]. Specifically, The GN particles directionally delivered and slowly released Adr molecules to the tumor sites. Upon laser treatment, more Adr molecules were released from the nanoparticles, resulting in a higher Adr concentration in the tumor tissues than that by administration of free Adr. As a result, the Adr-GNs+Laser and Adr-GNs+siRap2b-GNs+Laser treatments exhibited a higher tumor killing effect than others. Furthermore, in contrast to Adr-GNs and Adr-GNs+Laser treated mice, additional siRap2b-GNs resulted in a significant reduction in the tumor growth, indicating an enhancing role of siRap2b in the anticancer therapeutics.

In the current research, we studied the role of siRap2b in the improvement of anticancer capability of Adr in a GN-based drug/gene co-delivery system. However, additional studies are needed before potential clinical trials, such as on immunogenicity and pharmacokinetics parameters, both of which are important indexes to estimate the applicability of nanoparticles for clinical practice. Moreover, our data indicated that without laser exposure, the loaded Adr and siRNA also showed a slow release from the GNs. Before we translate this nanoformulation into clinical practice, we should reduce this off-target background release. Besides intrinsic and passive targeting of nanoparticles via an EPR effect, a critical way is via fast and active tumor targeting. Therefore, in the future, we will modify and conjugate some tumor antigen specific ligands, such as EGFR antibody and RGD peptide, to the surface of GNs for an enhanced active targeting [41, 42]. Due to the ligand-driven active targeting, the modified GNs will accumulate in tumors faster than that passively driven by an EPR effect [27] and thus lead to a less off-target drug release. Besides, many other technologies and methods can be adapted for drug loading or modification of GNs to optimize the nanoformulation for an appropriate administration and delivery [43].

## MATERIALS AND METHODS

### Materials and measurements

Lipofectamine^®^ 2000 (lip2000) was from Thermo Fisher Scientific Inc. Adr, NaBH_4_, AgNO_3_, HAuCl_4_, MTT, sodium citrate, hydroxylamine hydrochloride (NH_2_OH·HCl), McCoy’5A and MEM medium, Protease Inhibitor Cocktail, and other reagents were bought from Sigma-Aldrich. Diethylpyrocarbonate (DEPC) was a product from Amresco. Fetal bovine serum (FBS) was from Hyclone. HiFiScript cDNA Synthesis Kit and RIPA Lysis Buffer (Cat No. CW2334) were purchased from CWBiotech Inc., China. EvaGreen 2×qPCR MasterMix were from Applied Biological Materials Inc., Canada. All antibodies were from Abcam. MPEG-SH-2000 was purchased from Laysan Bio, Inc. SiRap2b (sense, 5′-GACGAGCUAUUUGCCGAGATT-3′) was synthesized by Shanghai GenePharma Technology Co. Ltd., China. The custom-synthesized siRNA was functionalized with a sulfhydryl group at the 5′ end of the sense chain and the thiolated siRNA duplex was labeled with Rh123 at the 3′ end of the sense chain. The SH-5′-siRap2b-3′-Rh123 was also synthesized by Shanghai GenePharma Technology Co. Ltd.

Fluorescence images were captured under a laser scanning confocal microscope (Leica TCS SP8 STED). A UV absorption spectrum was measured with a microplate reader (TECAN Infinite M200 Pro). The image of nanoparticles was recorded using a CM100 transmission electron microscope (Philips, Netherlands). SiRNA concentration was determined using a micro-spectrophotometer (Nano-100, Shanghai, China). Laser irradiation was performed using a diode laser system (BWT, Beijing, China). Western-blot images were obtained from a Tanon-5200 Chemiluminescence Apparatus (Tanon Science & Technology Co. Ltd., Shanghai, China).

The primers used in the qRT-PCR were synthesized by GENEWIZ Suzhou, China. The PCR sequences were listed below:

Rap2b (human, NM_002886.3): forward primer 5′-GAC GTC GGC CAA AAA CAA A-3′; reverse primer 5′-CGC ACG ATC TCG GCA AAT-3′.

28S (human, NR_003287.2): forward primer 5′-GGC GAA GCC AGA GGA AAC T-3′; reverse primer 5′-GAC GAC CGA TTT GCA CGT-3′.

### Cell lines and animal models

HCT116 cells were kindly provided by Cell Bank, Chinese Academy of Sciences. MCF-7 cells were a gift from Dr. Renqing Feng of Peking University. The culture medium for HCT116 and MCF-7 cells was McCoy's 5A and MEM medium supplemented with 10% FBS, respectively. The cells were cultured in a humidified atmosphere containing 5% CO_2_ at 37°C.

All animal experiments were carried out in compliance with the Animal Management Rules of the Ministry of Health of the People's Republic of China. HCT116 cells (2.5 × 10^6^ per mouse) were subcutaneously injected into the upper right axillary fossa in the nude mice (Charles River Laboratories) aged 4∼6 weeks with a body weight of 18∼22 g. As the tumors grew up to a diameter of 0.3∼0.4 cm, the mice were used for treatment.

### Preparation of GNs

GNs were prepared by reducing HAuCl_4_ onto silver nanoparticle templates [12]. In brief, silver nanoparticles were prepared by adding 2 mM NaBH_4_ into a well stirred solution of 4 μM AgNO_3_ and 10 μM sodium citrate. The reaction produced a characteristic yellow color and was allowed to stir at 60°C for ≥ 2 h, followed by cooling to room temperature. Then, silver particle growth was initiated by adding 4 mM NH_2_OH·HCl to the resulting silver sol and continued to stir for 5 min. Next, 0.1 M AgNO_3_ was added to the sol and turned it dark yellow or orange. After stirring for ≥ 2 h (often overnight), the sol was heated to 60°C. Then, 0.5 mM HAuCl_4_ was added into the sol, followed by stirring for 1 h. GNs started to form in the sol via replacement chemistry. Once the reaction was complete, the sol was cooled down and silver chloride was allowed to precipitate. Next, the supernatant containing GNs was transferred to another vessel and stored at 4°C until use. GN concentration was calculated as 5 × 10^10^ mL^−1^, based on the method by Prevo, *et al*. [23].

Subsequently, the GNs were modified with MPEG-SH-2000 via thiol-Au bonds [44] for steric stabilization and *in vivo* circulation half-life enhancement [26, 27]. PEGylation were performed according to the published protocol with slight modifications [12]. In brief, the GNs (6 × 10^12^ mL^−1^) were added into an aqueous solution containing 3 × 10^−10^ M MPEG-SH-2000. The reaction proceeded overnight at room temperature. PEGylated GNs were purified by centrifugation at 14000 rpm for 20 min and the pellet was resuspended in deionized water. The process was repeated twice to remove unreacted MPEG-SH-2000 molecules.

### Adr and siRap2b loading onto GNs

Adr-GNs and siRap2b-GNs were prepared according to the reported methods with slight modifications [12, 45, 46]. In brief, Adr molecules were conjugated to GNs via amino-Au bonds formed between its active amino groups and gold nanoparticles. An aliquot of free Adr in water (0.35 mg, 0.1 mL) was added into an aqueous solution of PEGylated GNs (6 × 10^12^ particles, 1 mL), and the mixture was stirred at room temperature for 24 h. After centrifugation (14000 rpm for 20 min), the precipitate was washed with PBS and centrifuged. All supernatants were collected and pooled together. The amount of free Adr in the supernatant was determined by spectrophotometry at 480 nm. The loading efficiency (LE) of Adr was estimated using two methods. The first one indirectly measures attached Adr by determining the amount of unbound Adr in the supernatant according to equation 1: LE_indirect_ = (total amount of Adr used – amount of free Adr in the supernatant)/total amount of GNs. The second one directly quantifies attached Adr after extraction of Adr from dried GNs with DMSO according to equation 2: LE_direct_ = total amount of Adr extracted from GNs/total amount of GNs. Both calculations showed that, on average, 6 × 10^4^ Adr molecules were conjugated to the surface of each GN particle.

Similar to generation of Adr-GNs, siRap2b-GNs were produced by conjugating SH-5′-siRap2b-3′-Rh123 to GNs via thiol-Au bonds with a slight difference in reaction condition, that is, 40 μg of SH-5′-siRap2b-3′-Rh123 was added into 1 mL of a PEGylated GN solution containing 6 × 10^12^ particles in DEPC-treated water. The LE of siRap2b was quantified indirectly according to the equation: LE = (total amount of siRap2b used – amount of siRap2b in the supernatant)/total amount of GNs. In the end, about 200 siRNA molecules were loaded onto each GN particle.

### Measurement of laser-induced thermal effect

10^12^ GN particles were resuspended in 2 mL of McCoy's 5A medium in a centrifuge tube. The tube was placed in a 37°C water bath and then exposed to an 808 nm laser (2 W·cm^−2^). Solution temperature was monitored at different time point post irradiation. In the meanwhile, a same volume of GN-free McCoy's 5A medium was used as a negative control.

### Adr and siRap2b release from GNs

Two milliliters of Adr-GNs or siRap2b-GNs (10^12^ particles/mL) in each tube was placed in a 37°C water bath. The samples were divided into 2 groups: one was for irradiation, the other not. At predetermined time intervals, the samples were irradiated with an 808 nm laser (2 W·cm^−2^) for 1 min. The nanoparticle solution was centrifuged at 4000 rpm for 20 min and Adr or siRap2b in the supernatant was determined for analysis of the amount.

### Cellular uptake of Adr-GNs and siRap2b-GNs

HCT116 cells were seeded in confocal dishes (Φ35 mm) and incubated for 24 hours, then replaced with 1 mL of culture medium containing 3 × 10^11^ nanoparticles. After incubated for 0, 2, 4, 6, and 12 h, the cells were washed with PBS and then imaged under a laser confocal microscope. The cellular fluorescent intensity was analyzed using the built-in image analysis software LAS X and the mean value was calculated based on the data from 10 cells.

### Knockdown of Rap2b

Cancer cells (5 × 10^4^ per well) were seeded in a 12-well plate. One day later, the cells were incubated for 48 h with siRap2b-GNs (0.5 nM) carrying 100 nM siRap2b or siRap2b-lip2000 mixture containing 100 nM siRap2b. Untreated cells were used as a control. For further analysis of Rap2b knockdown in the presence of Adr, cancer cells were incubated with siRap2b-GNs or siRap2b-lip2000 for 40 h, followed by an additional incubation with 1 μM Adr for 8h. The cells treated with 1 μM Adr were used as a control in this context. Knockdown efficiency was assessed by qRT-PCR and western blotting.

### Reverse transcription and real-time PCR

Total RNA was isolated from the cells using Trizol reagent and dissolved in DEPC-treated water. Reverse transcription was performed with a HiFiScript cDNA Synthesis Kit. Real-time PCR was carried out in duplicates with a total volume of 10 μL using EvaGreen 2 × qPCR MasterMix. Reactions were run in a LightCycler^®^ 480 Real-time PCR system (Roche). The results were analyzed using the built-in software LightCycler^®^ 480 version 1.5. Rap2b mRNA levels were normalized to that of 28S. The relative Rap2b mRNA levels were calculated by comparing the normalized values to that of untreated cells, the value of which was set to 1.

### Western blotting

The cells were lysed in RIPA Lysis Buffer containing Protease Inhibitor Cocktail and 1 U/ml DNase I on ice for 20 min, followed by centrifugation at 12000 g for 10 min at 4°C. The lysate concentration was determined by the Bradford assay (Bio-Rad). Fifty micrograms of total protein was separated on a 15% SDS-polyacrylamide gel and transferred onto a nitrocellulose membrane (Bio-Rad). The membrane was blocked with 5% milk in PBST (1 × PBS with 0.1% Tween 20) at room temperature for 1 h, and incubated with primary antibody at 4°C overnight and secondary antibody at room temperature for 1 h. Subsequently, the membrane was developed in an UltraECL solution (YuanPinHao Bio, Beijing, China). Images were obtained from a Tanon-5200 Chemiluminescence Apparatus. For multiple detections, the membrane was stripped with Stripping Buffer (CWBiotech, China). The primary antibodies used in this study were against Rap2b and β-actin. The secondary antibody was HRP-labeled anti-mouse IgG.

### *In vitro* killing of cancer cells

Cell viability assay was carried out to evaluate the killing effect of drug-loaded GNs on HCT116 cells with or without irradiation. HCT116 cells (3000 per well) were seeded into a 96-well plate and cultured for 24 h prior to the following 7 groups of treatments:

Group 1: siRap2b-GNs+Adr-GNs+Laser. Adr-GNs (0.0183, 0.00914, 0.00457, 0.00228, 0.00114, 0.000571 nM) carrying corresponding concentrations of Adr (0.64, 0.32, 0.16, 0.08, 0.04, and 0.02 μg/mL, respectively) were added into the wells in quadruplicates. In addition, all wells were added with siRap2b-GNs (5 × 10^−4^ nM) carrying an average of 200 siRap2b/GN. Four hours later, GN accumulation reached a maximum in cancer cells (Figure 4). Then, the cells were exposed to an 808 nm laser (2 W·cm^−2^) for 1 min and cultured for an additional 44 h.

Group 2: siRap2b-GNs+Adr-GNs. Cells were treated according to the protocol described in Group 1 except without laser irradiation. Both Adr-GNs and siRap2b-GNs were co-cultured with the cells for a continuous 48 h.

Group 3: Adr-GNs+Laser. Cells were co-incubated with different concentrations of Adr-GNs which were consistent with those in Group 1. Free GNs (5 × 10^−4^ nM) were added into the wells to make total amount of GN particles consistent with those in Groups 1 and 2. Laser irradiation was performed according to the protocol in Group 1.

Group 4: Adr-GNs. Cells were treated with Adr-GNs for 48h using the protocol described in Group 3 except without a laser irradiation.

Group 5: Free Adr. Cells were treated for 48 h with different concentrations of free Adr (0.64, 0.32, 0.16, 0.08, 0.04, and 0.02 μg/mL).

Group 6: Free Adr+Free siRap2b. Cells were treated for 48h with 0.1 nM free siRap2b plus various concentrations of Adr described in Group 5.

Group 7: Free Adr+siRap2b-lip2000. The protocol was similar to that carried out in Group 6 except that the siRap2b was premixed with 2 volumes of lip2000.

Group 8: Free GNs+Laser. In compliance with the final GN dosages in Groups 1–4, Free GNs (0.0188, 0.00964, 0.00507, 0.00278, 0.00164, 0.001071 nM) were added into the wells followed by a 48 h co-incubation. Laser irradiation was performed according to the protocol in Group1.

Group 9: Free GNs. Cells were treated with Free GNs for 48 h using the protocol described in Group 8 except without a laser irradiation.

The concentrations of Adr were consistent in Groups 1–7, and those of GNs were consistent in Groups 1–4, 8, and 9. Upon treatment, MTT solution was added into each well. After an additional 4 h incubation, the absorbance of each well was measured at 490 nm with a microplate reader.

### *In vivo* therapeutic efficacy

HCT116 tumor-bearing nude mice were randomly divided into 6 groups (*n* = 6 per group). The mice were treated with 0.2 mL agents via tail vein injection every three days for 15 days: (Group A) Saline (control group); (Group B) Free Adr (3 mg/kg); (Group C) Adr-GNs (0.0857 nmol/kg) carrying Adr (3 mg/kg) plus free GNs (1.25 nmol/kg); (Group D) Same to Group C; (Group E) Adr-GNs (0.0857 nmol/kg) plus siRap2b-GNs (1.25 nmol/kg) carrying siRap2b of 0.25 μmol/kg; and (Group F) Same to Group E. The total amount of GN particles was identical in Groups C, D, E, and F. In addition, at 6 h post injection [25], all tumor sites in Groups D and F were exposed to an 808 nm laser (2 W·cm^−2^) for 1 min. Therapeutic efficacy of each drug/laser treatment was evaluated by measuring tumor volume and body weight of each mouse every three days. At the end of 15 d treatment period, all mice were sacrificed and tumors were resected for photography.

### Statistical analysis

Numerical data were expressed as mean ± standard deviation. The significance of the difference between the mean values of two groups was evaluated with Student *t-test*. Differences were considered statistically significant at *P* < 0.05 (*) and *P* < 0.01 (**).

## Abbreviations

Adr: adriamycin
siRap2b: Rap2b siRNA
GNPs: gold nanoparticles
SNAs: spherical nucleic acid nanoparticle conjugates
EPR: enhanced permeability and retention
NIR: near infrared ray
GN: gold nanoshell
siRap2b-GNs: Rap2b siRNA conjugated gold nanoshells
Adr-GNs: adriamycin conjugated gold nanoshells
Rh123: Rhodamine 123
Lipofectamine^®^ 2000: lip2000
MTT: methylthiazolyldiphenyl-tetrazolium bromide
FBS: Fetal bovine serum
qRT-PCR: quantitative real-time polymerase chain reaction
MDR: multidrug resistance
LE: loading efficiency
DEPC: diethylpyrocarbonate
